# Fatty Acid Biosynthesis Pathways in *Methylomicrobium buryatense 5G(B1)*

**DOI:** 10.3389/fmicb.2016.02167

**Published:** 2017-01-10

**Authors:** Aleksandr Demidenko, Ilya R. Akberdin, Marco Allemann, Eric E. Allen, Marina G. Kalyuzhnaya

**Affiliations:** ^1^Department of Biology, San Diego State University, Campanile DriveSan Diego, CA, USA; ^2^Scripps Institution of Oceanography, University of California San Diego, Gilman DriveLa Jolla, CA, USA

**Keywords:** methane valorization, methanotrophs, fatty acid metabolism, farE, fatty acid elongation

## Abstract

Methane utilization by methanotrophic bacteria is an attractive application for biotechnological conversion of natural or biogas into high-added-value products. Haloalcaliphilic methanotrophic bacteria belonging to the genus *Methylomicrobium* are among the most promising strains for methane-based biotechnology, providing easy and inexpensive cultivation, rapid growth, and the availability of established genetic tools. A number of methane bioconversions using these microbial cultures have been discussed, including the derivation of biodiesel, alkanes, and OMEGA-3 supplements. These compounds are derived from bacterial fatty acid pools. Here, we investigate fatty acid biosynthesis in *Methylomicrobium buryatense 5G(B1)*. Most of the genes homologous to typical Type II fatty acid biosynthesis pathways could be annotated by bioinformatics analyses, with the exception of fatty acid transport and regulatory elements. Different approaches for improving fatty acid accumulation were investigated. These studies indicated that both fatty acid degradation and acetyl- and malonyl-CoA levels are bottlenecks for higher level fatty acid production. The best strain generated in this study synthesizes 111 ± 2 mg/gDCW of extractable fatty acids, which is ~20% more than the original strain. A candidate gene for fatty acid biosynthesis regulation, *farE*, was identified and studied. Its deletion resulted in drastic changes to the fatty acid profile, leading to an increased pool of C18-fatty acid methyl ester. The FarE-regulon was further investigated by RNA-seq analysis of gene expression in *farE*-knockout mutants and *farE*-overexpressing strains. These gene profiles highlighted a novel set of enzymes and regulators involved in fatty acid biosynthesis. The gene expression and fatty acid profiles of the different *farE*-strains support the hypothesis that metabolic fluxes upstream of fatty acid biosynthesis restrict fatty acid production in the methanotroph.

## Introduction

Methane bioconversion by methanotrophic bacteria is attracting attention from biotechnologists because of its inexpensiveness and the abundance of natural gas as its potential feedstock. However, economically effective examples of such bioconversions have been limited to just the production of single-cell protein and polyhydroxybutyrate products (Levett et al., 2016; Strong et al., 2016). Advancement in this area is limited by the paucity of approaches to manipulate the methanotroph genome and the even more limited knowledge of the genomic targets to be manipulated. We and others are developing genomics and genetic tools for methanotroph genome manipulation (Ojala et al., 2011; Puri et al., 2015; Henard et al., 2016). In this work we focus on identifying novel genomic targets—specifically, we studied fatty acid (FA) biosynthesis/degradation pathways and regulation as a means to increase cellular lipid content. Total FAs extracted from bacteria have been shown to be valuable precursors for production of liquid transportation fuels (Lennen and Pfleger, 2012). Furthermore, the interest in methanotrophic microbes as methane-driven microbial factories for the production of a variety of FA-based products, ranging from industrial surfactants and detergents to cosmetic carotenoid-based products and OMEGA supplements, is on the rise. Methanotrophic bacteria produce high levels of FAs for the synthesis of intracytoplasmic membranes (ICM, Patt and Hanson, 1978; Jahnke and Nicholst, 1986; Sessions et al., 2002). However, the metabolic pathways for FA biosynthesis have never been thoroughly investigated.

**Figure 1:**
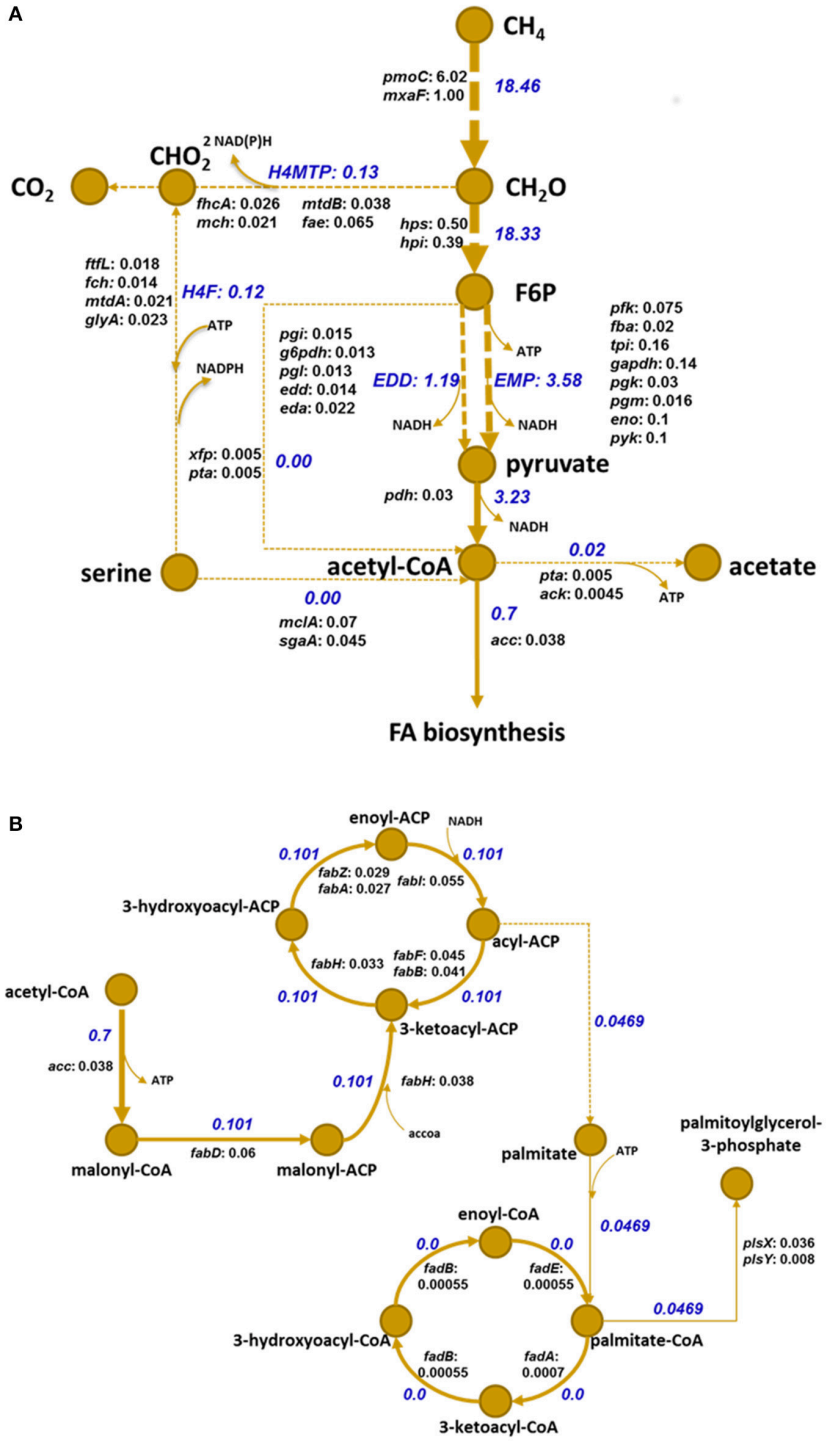
**Metabolic pathways for methane oxidation (A)** and FA synthesis **(B)** in *M. buryatense 5G(B1)* based on a gene inventory study, transcriptomic data analysis, and genome-scale modeling (De la Torre et al., 2015). Numbers in blue represent that particular reaction's fluxes according to the developed genome-scale model (De la Torre et al., 2015); numbers in black after gene names denote abundances of respective transcript normalized to an abundance of methanol dehydrogenase. Gene names coding enzymes of the methane assimilation pathways are extracted from BioCyc Database Collection (http://biocyc.org/). Enzyme EC numbers and corresponding gene IDs are listed in Table S5. Methane utilization starts from oxidation to methanol by methane monooxygenase enzyme in periplasm; methanol is then metabolized to formaldehyde by a periplasmic pyrroloquinoline quinone-linked methanol dehydrogenase. Formaldehyde is either oxidized to CO_2_ or assimilated in cytoplasm through ribulose monophosphate (RuMP) and EMP/EDD (Embden-Meyerhof-Parnas/Entner–Doudoroff) pathways **(A)**, fraction of which are then converted through pyruvate to acetyl-CoA, a precursor for FA synthesis **(B)**. Designations: CH_4_, methane; CH_2_0, formaldehyde; CHO_2_, formate; H4MTP, tetrahydromethanopterin pathway; H4F, methylene tetrahydrofolate pathway; CO_2_, carbon dioxide; f6p, fructose 6-phosphate; acCoA, acetyl-CoA; NADH, nicotinamide adenine dinucleotide reduced; NADPH, dihydronicotinamide adenine dinucleotide phosphate reduced; ATP, adenosine-triphosphate.

*Methylomicrobium buryatense 5G(B1)*, a gammaproteobacterial methanotrophic bacterium, can use methane as a sole carbon and energy source. A set of unique properties of the strain, including its fast growth rate, its ability to grow over a broad range of salinity and pH, and the availability of a draft genome, a validated flux balance model, and a set of genetic tools, make it an ideal model for studying lipid biosynthesis (Kaluzhnaya et al., 2001; Khmelenina and Beck, 2013; De la Torre et al., 2015; Puri et al., 2015; Henard et al., 2016). The metabolic pathways for methane utilization in *M. buryatense 5G(B1)* have been recently refined (De la Torre et al., 2015; Gilman et al., 2015). Methane utilization starts with the oxidation of methane to methanol by a particulate or soluble methane monooxygenase enzymes (Kaluzhnaya et al., 2001); methanol is then converted to formaldehyde by PQQ-dependent methanol dehydrogenases, a two subunit Ca-containing enzyme or a one subunit La-induced enzyme (Chu and Lidstrom, 2016; Chu et al., 2016). Formaldehyde is either oxidized to CO_2_ to supply energy or assimilated into sugars through a ribulose monophosphate (RuMP) pathway, a fraction of which is then converted through pyruvate to acetyl-CoA (Figure 1A), a building block for FA synthesis (Figure 1B). Similar to other methanotrophic bacteria, *M. buryatense 5G(B1)* produces significant amounts of ICMs, which can occupy up to 60% of its cellular volume (Collins and Kalyuzhnaya, unpublished data). Methanotroph membranes are composed of phosphatidylethanolamine- (63–95%), phosphatidylglycerol- (2.5–18%), and phosphotidylserine- (2.2–7.6%) linked C_16_ or C_16:1_ FAs (Kaluzhnaya et al., 2001; Gilman et al., 2015). Here, we present a genome-based reconstruction of the FA biosynthesis pathways in *M. buryatense 5G(B1)*. Assumptions from the analysis of genomic data were further supported by gene expression data and mutagenesis. A detailed search within the *M. buryatense 5G(B1)* genome revealed several candidate gene targets for strain engineering efforts to improve production of bulk FA-based chemicals.

## Materials and methods

### Strains and genetic manipulations

Strains and genetic constructs used in this study are listed in Table 1. Strain AP18 was chosen as the base strain for most of the genetic modifications described here. The strain descends from the *M. buryatense 5G(B1)* strain, a lab-adapted variant of wild type *M. buryatense* 5G. The strain is resistant to rifampicin, has improved transformation efficiency, and lacks the 76 kB plasmid, the glycogen synthase genes *glgA1* and *glgA2*, and the sucrose-phosphate synthase (*sps)* gene (Puri et al., 2015). These modifications resulted in a very modest increase in the FA pool as well as an inability to produce glycogen and/or sucrose. So far no significant impacts on cell growth and methane consumption have been observed. The strain AP18 and plasmid constructs used to create acetate kinase-deleted and acetyl-CoA carboxylase-overexpressing mutants were kindly provided by the Lidstrom Laboratory (University of Washington).

**Table 1:** **List of strains and plasmids used in this study**.

**Strain or Plasmid**	**Description**	**Reference source**
*E. coli* DH5α	F– Φ80lacZΔM15 Δ(lacZYA-argF) U169 recA1 endA1 hsdR17 (rK–, mK+) phoA supE44 λ– thi-1 gyrA96 relA1	
*E. coli* DH5α	Strain with chromosomally integrated conjugal RP4 transfer functions for biparental conjugation	
AP18	*M. buryatense 5G(B1)*c ΔglgA1 ΔglgA2 Δsps (WP_017842105.1, WP_017842106.1, WP_017840914.1), cured of native plasmid	Puri and Lidstrom, unpublished data
AP18Δack	AP18 Δack (WP_017840011.1)	This study
AP18ΔfadABE	AP18 ΔfadA (WP_017841934.1) ΔfadB (WP_017841933.1) ΔfadE (WP_017841932.1)	This study
AP18::acc	AP18 with pAWP145 containing accA (WP_017842193.1), accB (WP_017839152.1), accC (WP_017839151.1), and accD (WP_017839055.1)	This study
AP18Δack::acc	AP18 Δack with pAWP145	This study
AP18ΔfadABE-ack::acc	AP18 ΔfadA ΔfadB ΔfadE Δack with pAWP78 containing accA, accB, accC, and accD	This study
AP18ΔfarE	AP18 ΔfarE (WP_017839568.1)	This study
AP18::farE	AP18 with pAWP78 containing farE	This study
AP18::fabB	AP18 with pAWP78 containing fabB (WP_017839697.1)	This study
AP18::acp	AP18 with pAWP78 containing acpP (WP_014148504.1)	This study
AP18::fabB::acp	AP18 with pAWP78 containing fabB and acpP	This study
pCM433	Plasmid for making unmarked mutants	Marx, 2008
pCM184	Plasmid for making kanamycin-resistant mutants	Marx and Lidstrom, 2002
pAWP78	IncP-based broad host range plasmid for gene overexpressions	Puri et al., 2015
pAWP145	Tetracycline-inducible construct for overexpression of AccA (WP_017842193.1), AccB (WP_017839152.1), AccC (WP_017839151.1), and AccD (WP_017839055.1)	Puri and Lindstrom lab, UW
pCM433Δack	Variant of pCM433 containing flanks to knock out Sck	Lindstrom, UW
pCM433ΔfadABE	Variant of pCM433 containing flanks to knock out FabA, FadB, and FadE	This study
pCM184ΔfarE	Variant of pCM184 containing flanks to knock out FarE	This study
pAWP78::farE	Variant of pAWP78 containing FarE	This study
pAWP78::fabB	Variant of pAWP78 containing FabB	This study
pAWP78::acpP	Variant of pAWP78 containing ScpP	This study
pAWP78::fabB::acpP	Variant of pAWP78 containing FabB and ScpP	This study

*M. buryatense* strains were grown on methane as described (Ojala et al., 2011), with some modification of the growth medium (Table S1). *Escherichia coli* strains were grown on Luria-Bertani media supplemented with kanamycin (100 μg/ml) and ampicillin (100 μg/ml). Genetic manipulations with *M. buryatense* were done as described (Puri et al., 2015). For unmarked gene deletions, the pCM433kanT plasmid carrying ~600-bp of sequences flanking the to-be-deleted genes was introduced. After conjugation, single-crossover kanamycin-resistant *M. buryatense* clones were plated on rifampicin to counter-select against *E. coli*. Then, to select for Kan-sensitive double crossover clones with deleted genes of interest, single-crossover clones were passaged on plates with 2.5% sucrose and the resulting colonies were PCR-genotyped for the absence of the gene of interest followed by sequencing. For overexpression of genes, the pAWP78 plasmid was used as a vector. All genes were cloned under the *tac*-promoter, which provides strong constitutive expression in *M. buryatense* (Puri et al., 2015). The nucleotide positions of the sequences cloned into plasmids are outlined in Table S2.

### Induction of acetyl-CoA carboxylase

The acetyl-CoA carboxylase expression system (pAWP78::accABCD) was obtained from Lidstrom and Puri. The construct includes the native acetyl-CoA carboxylase genes (subunits A, B, C, and D) cloned into pAWP78 under the tetracycline-inducible pTet promoter (Puri, unpublished data). The plasmid was transformed into *E. coli* S17–1 and transferred into the 5GB1C, AP18, AP18 Δack, AP18ΔfadABEΔack, abd AP18ΔfadABE backgrounds via conjugation. Cells were grown until mid-exponential phase (at OD ~0.5). Each cell line was represented by biological (3) and technical (2 per biological replicate) replicates. Expression of acetyl-CoA carboxylase was induced by addition of anhydrotetracycline (1 μM final) followed by a 6-h incubation before collection of cells by centrifugation.

### FA methyl ester (FAME) analyses

Fifty milliliters of cell cultures grown at 30°C with 50 ml of methane to OD_600_ ~1 were collected by centrifugation, washed twice with 10 mM Tris-Cl (pH8.0) and 50 mM of NaCl, and lyophilized. Biological replicates (*n* = 2 ÷ 10) were submitted to Matrix Genetics (Seattle, WA, http://matrixgenetics.com) for FAME derivatization and GC-MS analysis.

### RNA sequencing

Strains (i) AP18 (ii) AP18ΔfabR, and (iii) AP18::pAWP78 (fabR) were grown in duplicate in a 50 ml volume at 30°C with shaking with methane. RNA from pelleted cells was extracted according to (Griffiths et al., 2000) and treated with PureLink DNaseI (ThermoFisher Scientific) according to the manufacturer's instructions. Samples were sequenced on Illumina HiSeq4000 with ~50 million/sample SR50 reads by IGM Genomics Center, University of California, San Diego.

### Construction and refinement of the flux balance model for *M. buryatense 5G(B1)* in COBRA toolbox

To convert the flux balance model for *M. buryatense 5G(B1)* into the COBRA Toolbox format (Schellenberger et al., 2011) we used the published genome-scale metabolic network of this strain (De la Torre et al., 2015) developed in Pathway Tools (Karp et al., 2010). Subsequently, all zero-flux reactions were removed from the model. The information about zero-flux reactions was extracted from the solution file of the Pathway Tools. We manually extended the COBRA model by adding exchange rates for both nutrients and secreted metabolites as well as by the biomass equation. Moreover, we used the Paint4Net tool (Kostromins and Stalidzans, 2012) installed with the COBRA Toolbox to define dead-ends in the model network and to add corresponding exchange rates for them. To standardize reaction and metabolite IDs, we used abbreviations according to BiGG Models ID Specification and Guidelines (King et al., 2016) and KEGG abbreviations for enzymatic reactions for which we could not find corresponding BIGG reactions. To link genes with certain enzymatic reactions we turned to account NCBI Reference Sequence annotation: NZ_KB455575. Moreover, we added missing formulas for metabolites and checked out the model in COBRA Toolbox to ensure all reactions were mass balanced. The model was also extended by the addition of enzymatic reactions (EC 3.1.2.2; 1.3.8.-; 4.2.1.17; 1.1.1.35; 2.3.1.16) for FA degradation via the β-oxidation pathway and a reaction catalyzed by phosphoketolase (EC 4.1.2.22). Flux balance analysis (FBA) was performed by solving the Linear Programming problem for biomass optimization on the basis of GNU Linear Programming Kit (GLPK) (http://www.gnu.org/software/glpk/) solver in MATLAB. The converted model for *M. buryatense 5G(B1)* in COBRA format is available on the web-site: http://sci.sdsu.edu/kalyuzhlab/.

### Computational analysis of the RNA-Seq data

The genome sequence and annotation files of *M. buryatense 5G(B1)* were obtained from NCBI (RefSeq accession number GCF_000341735.1). Reads were aligned to the genome sequence using the Rockhopper 2 software system (Tjaden, 2015) with default settings. It is worth noting that the tool reports integral values for analyzed transcriptomic characteristics, even if the underlying value is a decimal. If a read aligns partially to a transcript, it will contribute a fractional amount to the raw count. Rockhopper 2 normalizes each RNA-seq data set using upper quartile normalization, because the normalization procedure of the mapped read counts is an essential preprocessing step for differential expression detection (Bullard et al., 2010). To quantify transcript abundance levels, Rockhopper 2 employs a modified RPKM value based on more robust normalizer of upper quartile transcript expression. The normalized data were used to compare gene expression in the wild type AP18 strain vs. the overexpressed *farE* and Δ*farE* strains using the built-in algorithm of DESeq (Anders and Huber, 2010). To determine whether a transcript shows differential expression in data from two any conditions we used a threshold (*q* < 0.05) of *q*-values that are corrected *p*-values to control the false discovery rate using the Benjamini-Hochberg procedure (Benjamini and Hochberg, 1995). Appropriateness of the *q*-value threshold <0.05 was verified with a set of housekeeping genes and genes encoding key C1-enzymes, and those showed no differential expression [for example: 3-hexulose-6-phosphate synthase, q(AP18 vs. AP18ΔfarE) = 0.28, q(AP18 +pAWP78 vs. AP18::farE) = 0.53; pyruvate carboxylase, q(AP18 vs. AP18ΔfarE) = 0.41, q(AP18 +pAWP78 vs. AP18::farE) = 0.64; arginyl-tRNA-protein transferase, q(AP18 vs. AP18ΔfarE) = 1, q(AP18 +pAWP78 vs. AP18::farE) = 1].

## Results

### Lipid biosynthesis and degradation: *M. buryatense 5G(B1)* genome mining

Methane metabolism in the *M. buryatense 5G(B1)* strain is outlined in Figure 1A. The automatic annotation of the genome predicts a typical Type II FA biosynthesis pathway. The FA-biosynthesis genes form several clusters: (1) a *plsXfabHDGacpfabF*-cluster, which includes genes for a fatty acid/phospholipid synthesis protein, 3-oxoacyl-ACP synthase III, malonyl-CoA-ACP transacylase, 3-oxoacyl-ACP reductase, acyl carrier protein (ACP), and 3-oxoacyl-ACP synthase II. The cluster also includes a hypothetical protein and a 50S ribosomal protein L32 gene upstream of *plsX*. The organization of this cluster is strongly conserved among all methanotrophic bacteria; (2) a *fabAB*-cluster encoding 3-oxoacyl-ACP synthase I and beta-hydroxydecanoyl thioester dehydrase; (3) a *fabGFZ/A cluster*, encoding 3-oxoacyl-ACP synthase II, 3-oxoacyl-ACP reductase, and beta-hydroxyacyl-ACP dehydratase; and (4) an *accCB*-gene cluster encoding biotin carboxylase and biotin carboxyl carrier protein (BCCP). Genes encoding the acetyl-coenzyme A carboxylase carboxyltransferase alpha subunit (*accA*) and beta subunit (*accD*), a second copy of the acetyl-CoA carboxylase biotin carboxylase subunit (*accC*), an additional (3R)-hydroxymyristol ACP dehydratase (*fabZ*), and two genes for ACP are located at sites distant from each other and other FA-biosynthesis genes.

Synthesis of phospholipids in *M. buryatense 5G(B1)* appears to follow the *E. coli* paradigm where a phosphatidic acid precursor is synthesized from glycerol 3-phosphate and FA precursors (Yao and Rock, 2012). However, no *plsB* (Glycerol-3-phosphate acyltransferase) homologs were identified within the *M. buryatense 5G(B1)* genome. Instead, homologs of *plsX* and *plsY* were found. *PlsXY* has been shown to preferentially utilize acyl-acyl carrier protein and not acyl-CoA as an acyl donor (Lu et al., 2006). A set of phospholipid-modifying enzymes whose homologs have been shown to act on phospholipids post-synthetically were found in the genome. The first of these enzymes is a membrane-bound desaturase, DesC, which shows about 60% identity to other well-characterized desaturases that catalyze the oxygen-dependent introduction of a double bond at the Δ9 position of saturated FAs, particularly stearic acid (Zhu et al., 2006; Parsons and Rock, 2013). Additionally, *M. buryatense 5G(B1)* contains two copies of *cfa*, a cyclopropane FA synthase, which uses S-adenosyl methionine to generate a cyclopropyl group at a double bond position of a FA (Grogan and Cronan, 1997). Cyclopropane FAs have been previously observed in other methanotrophic bacteria (*Methylococcus capsulatus, Methylobacterium organophilum)* during growth on methane and during oxygen limitation (Patt and Hanson, 1978; Jahnke and Nicholst, 1986). An intriguing aspect of these lipid-modifying enzymes in *M. buryatense 5G(B1)* is that both copies of *cfa* and *desC* are clustered together as part of a larger operon. Additionally the operon appears to encode a histidine sensor kinase, a lipase/esterase, and a sterol reductase gene. The physiological significance of this is unclear; however, it is tempting to speculate that these various activities are genetically linked as an operon for a coordinated membrane remodeling response in *M. buryatense 5G(B1)*. The *M. buryatense 5G(B1)* genome lacks free fatty transport gene, *fadL*, but contains an acyl-CoA synthetase gene, *acsA*, which implies that *M. buryatense 5G(B1)* cannot transport fatty acids. However, the rest of genes, *fadA, fadB*, and *fadE*, responsible for the degradation and recycling of FAs via β-oxidation, were identified. The presence of the PlsXY pathway for phospholipids biosynthesis and the lack of a FadD homolog suggest that *M. buryatense 5G(B1)* is not capable of incorporating exogenous fatty acids into its phospholipid membranes.

Neither fabR*/desT* nor *fadR* (a known transcriptional regulators of fatty acid biosynthesis/degradation in *E.coli*) homologs were detected. The lack of any recognizable transcriptional regulatory factors is unique and could imply that FA biosynthesis in *M. buryatense 5G(B1)* is being regulated by an as-yet-to-be discovered mechanism. It seems unlikely that FA biosynthesis in methanotrophs is not regulated as formation of ICMs is commensurate with an increase of FA content per cell (Gilman et al., 2015).

### Determining control points in fatty biosynthesis

The overreaching goal of the study was to identify targets to manipulate fatty acid biosynthesis in methanotrophs. The FBA suggests that about 7.5% of methane carbon enters the fatty acid biosynthesis pathway. According to genomic data, *M. buryatense 5G(B1)* possesses three different metabolic routes for production of acetyl-CoA from single carbon compounds with the main reaction contributing to acetyl-CoA formation predicted to be pyruvate dehydrogenase (Figure 1A). The FBA predictions are consistent with published enzymatic evidence and transcriptomic data (Figure 1; Kalyuzhnaya et al., 1999; De la Torre et al., 2015), as well as with transcriptomic data generated in this study.

**Figure 2:**
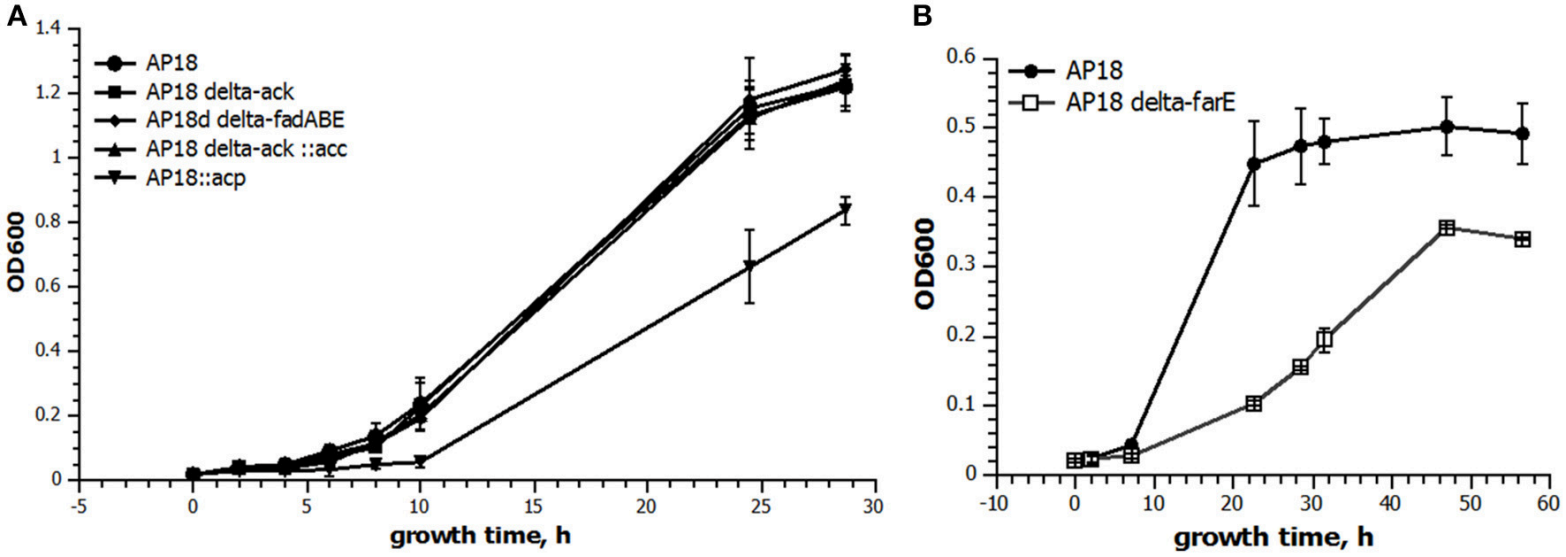
**Growth curves of select strains**. Twenty milliliters of cultures were grown in serum bottles with 50 ml methane at 30°C with shaking for the indicated time. The data are presented as the mean ± *SD* (*n* = 3). **(A)** Comparison of growth rates of the original AP18 (circles) and AP18Δack (squares), AP18ΔfadABE (diamonds), AP18Δack::acc (triangles), and AP18::acp (inverted triangles). **(B)** AP18 (filled circles) vs. AP18ΔfarE (open squares).

**Figure 3:**
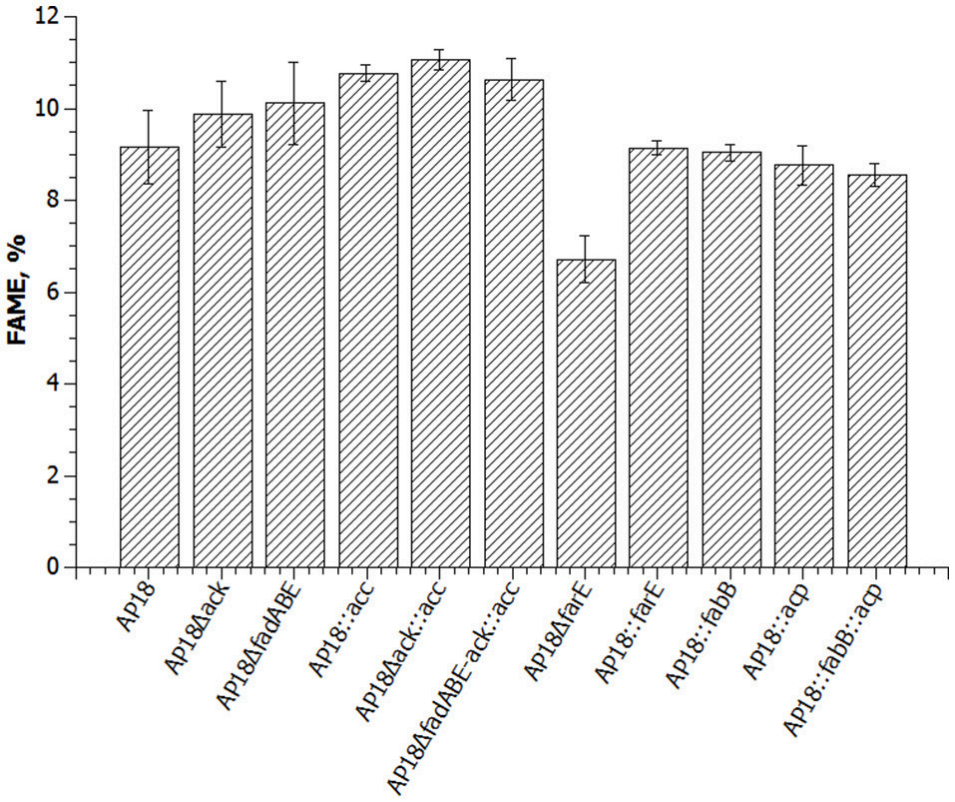
**Graphic presentation of FAME levels in the same strains as in Table 2 (percent = FAME/dry cell weight)**.

ACP is one of the key proteins in FA biosynthesis. In the *M. buryatense 5G(B1)* genome there are three genes encoding ACP of which only one is highly expressed (**Table 4**). The differential regulation of ACP expression raised a question about whether *M. buryatense 5G(B1)* FA metabolism is saturated with ACP or whether overproduction of ACP would stimulate FA synthesis. If ACP is a limiting factor, its overexpression would lead to an overall increase of the FA production. Alternatively, adverse effects on cell physiology might be observed if cells do not tolerate higher ACP levels. To distinguish between these hypotheses, we chose to overexpress the highly expressed *acp* gene (WP_014148504.1). An ACP-overexpressing plasmid was constructed and introduced into the AP18 strain. Unlike most other strains tested in this study, the ACP-upregulated strain showed growth defects (Figure 2) and no signs of increased FA production (Table 2, Figure 3). Addition of FabB to the overexpressing ACP strain did not enhance its FAME content. Thus, higher levels of ACP production seem to be toxic for the methanotrophic culture, as seen for *E. coli* (Keating et al., 1995).

**Table 2:** **FA methyl ester (FAME) content of the strains used in this study (percent = FAME/dry cell weight)**.

**Strain**	**FAME %**	**Increase, folds over AP18**
AP18	9.17±0.80	1.00
AP18Δack	9.88±0.72	1.08
AP18ΔfadABE	10.12±0.89	1.10
AP18::acc	10.77±0.18	1.17
AP18Δack::acc	11.07±0.22	1.21
AP18ΔfadABEΔack::acc	10.63±0.45	1.16
AP18ΔfarE	6.71±0.51	0.73
AP18::fare	9.14±0.15	1.00
AP18::fabB	9.04±0.19	0.99
AP18::acp	8.76±0.43	0.96
AP18::fabB::acp	8.54±0.25	0.93

To determine whether FA biosynthesis in methanotrophs is limited by the elongation step, ketoacyl-ACP synthases (*fabB*) overexpression was examined. No changes in either the amount of FAME or composition of FAME were observed (Table 2, Figure 3). In order to define the role of β-oxidation in methanotrophic FA metabolism, the entire cluster of *fadA, fadB*, and *fadE* genes was deleted. The Δ*fad(ABE)* mutant did not show significant changes in growth characteristics (Figure 2); furthermore, the Δ*fad(ABE)*-mutant cells had higher levels *of* FAs (10% increase compared to original AP18 strain; Table 2, Figure 3).

### Improving carbon flux into fatty acid building blocks: acetyl- and malonyl-CoA

Having established that neither ACP nor elongation of FA synthesis are limiting steps in FA accumulation, we then tested the possibility that directing carbon flux into acyl-CoA and malonyl-CoA pools would have a positive impact on total FA accumulation. *M. buryatense 5G(B1)* cells excrete acetate (~100 μmol gCDW^−1^) and formate (~500 μmol gCDW^−1^) during growth on methane (Gilman et al., 2015). The concentrations of excreted organic acids are not high; however, the leak of C_2_-compounds might indicate an additional loss of carbon needed for FA biosynthesis. In order to increase carbon flow into FA biosynthesis two additional modifications were made: deletion of acetate kinase (Δ*ack*) and overexpression of acetyl-CoA carboxylase (*acc*). It should be mentioned that the overexpression of acetyl-CoA carboxylase (*accABCD*) in the *M. buryatense* 5G(B1) strain did not show a statistically significant increase (Puri and Lidstrom, unpublished data). Here, we tested acc-overexpression in different genetic backgrounds. The AP18Δ*ack* and overproducing Acc strains were found to produce 99 ± 7 and 108 ± 2 mg/gDCW of FAME, respectively (Table 2, Figure 3). Moreover, when combined together in the same strain, the effects of both mutations were additive resulting in the highest FA levels (111 ± 2 mg/gDCW, ~20% more than in the original AP18) achieved. Deletion of *fadABE* genes in AP18Δ*ack*::*acc* background did not increase FAME levels.

### Regulation of fatty acid biosynthesis in *M. buryatense 5G(B1)*

The *M. buryatense 5G(B1)* genome does not contain proteins homologous to typical bacterial regulators of FA metabolism, such as *fabR, fadR*, or *desT*. However, an orf (WP_017839568) with a weak homology to the nucleotide-binding Maf family proteins was identified upstream of the *plsXfabHDGacpfabF*-cluster. Homologous genes were identified in eight methanotrophic genomes (*M. buryatense, Methylomicrobium alcaliphilum, Methylomicrobium marinus, Methylomicrobium luteus, M. sp MK WGS, M. sp.11b, Methylomicrobium methanica, and M. capsulatus)*, and in each case the gene located upstream of the FA biosynthesis genes. The conserved proximity of the fab-gene suggested it could be linked to FA metabolism. A WP_017839568 deletion mutant was generated and shown to have a severe growth defect (Figure 2). Cellular lipid content was also impaired: not only was the FAME content decreased but also there was an unusually high amount of C18:1 complemented by a decrease in C16:1 (Table 3). Reciprocal up-regulation (overexpression) of the WP_017839568 gene showed no growth defects and approximately the same FA profile as the initial strain (Table 3).

**Table 3:** **FA profile of original AP18 and strains with up- and down-regulated *farE***.

**Strain**	**Fatty acid, mg/gDCW**						
	**C14:0**	**C15:0**	**C16:1n7**	**C16:1n9**	**C16:0**	**C18:1**	**C18:0**
AP18, replicate 1	3.23	0.48	55.38	12.07	14.95	0.25	0.30
AP18, replicate 2	3.32	0.40	50.09	9.17	13.39	0.27	0.20
AP18ΔfaeR, replicate 1	0.00	0.00	23.49	6.24	8.70	24.06	0.97
AP18ΔfaeR, replicate 2	0.00	0.23	23.84	7.73	9.05	28.09	1.72
AP18::faeR, replicate 1	4.53	0.62	54.34	15.36	16.89	0.24	0.49
AP18::faeR, replicate 2	4.25	0.59	52.55	14.93	17.31	0.22	0.54

This striking phenotype of the WP_017839568-deleted strain suggested the possibility that this gene might be involved in the regulation FA synthesis, especially elongation of growing FA chains past 16 carbon atoms. In this regard, we proposed the name *farE* (for Fatty Acid Regulator of Elongation) for the WP_017839568 gene. In order to investigate the mechanism of such regulation, total RNA sequencing was performed (Table S3) for the following strains: (i) original AP18 strain (ii) AP18Δ*farE*, (iii) AP18::*farE*, and (iv) AP18::pAWP78 (empty plasmid). By comparison of AP18 to AP18Δ*farE*, ~1600 differentially expressed transcripts were identified. The majority of the differentially expressed genes are related to general metabolism and cellular functions (not shown), which could be explained by significant differences in growth rates between wild type and the *farE*-mutant. A subset of differentially regulated genes upon *farE* deletion is involved in FA biosynthesis or maintaining the acyl-CoA pool (Table 4). They include all three copies of the *acpP* gene present in *M. buryatense 5G(B1)* genome, two copies of the *fabF* and *fabA* genes, and the *fabG* and *fabD* genes. Two more insufficiently annotated genes included another copy of 3-ketoacyl-ACP synthase (WP_017841167.1) and a new thioesterase (WP_040575583.1, acyl-CoA thioester hydrolase). Considering their function in elongation of FA biosynthesis, upregulation of either 3-ketoacyl-ACP synthase (two copies of *fabF* and WP_017841167.1) could be a logical explanation for the elevated levels of the normally uncommon C18 FA in *M. buryatense 5G(B1)*. Upon deletion of *farE*, the Acp expression pattern changes so that all three *acpP* genes are slightly upregulated (1.32x, 1.75x, and 2.13x).

**Table 4:** **Differentially regulated genes of FA biosynthesis with down- and up-regulated *farE* compared to original AP18 (*q* < 0.05)**.

	**Gene ID**	**Gene function**	**Expression**		
			**AP18**	**AP18ΔfarE**	**AP18::farE**
fabA	WP_014149549.1	3-Hydroxyacyl-ACP dehydratase	89	102	200
fabA	WP_017839698.1	3-Hydroxydecanoyl-ACP dehydratase;	121	109	144
	WP_017841166.1	3-Hydroxyacyl-ACP dehydratase	39	60	31
fabG	WP_017841165.1	3-Ketoacyl-ACP reductase	33	109	38
–	WP_017841167.1	3-Ketoacyl-ACP synthase	29	61	32
fabF	WP_026130034.1	3-Oxoacyl-ACP synthase	166	162	291
fabF	WP_026130222.1	3-Oxoacyl-ACP synthase	40	54	38
–	WP_017840388.1	Acetyl-CoA carboxylase subunit alpha	73	82	56
acpP	WP_014148504.1	Acyl carrier protein	1136	1505	1733
acpP	WP_017841105.1	Acyl carrier protein	29	51	41
acpP	WP_017841106.1	Acyl carrier protein	62	132	65
–	WP_040575583.1	acyl-CoA thioester hydrolase	25	42	33
fabD	WP_017839572.1	Malonyl CoA-ACP transacylase	391	847	469
	WP_017840721.1	2-Isopropylmalate synthase	113	108	97
farE	WP_017839568	Fatty acid regulator of elongation	29	4	1658

*Calculated normalized transcript abundance levels are show*.

In order to further narrow down the list of potential candidates for explaining changes in the Δ*farE* mutant, we compiled a table (Table 5) including only those FA biosynthesis-related genes that are regulated in opposite directions in Δ*farE* and *farE* overexpression strains. In addition, genes differentially regulated in AP18 and AP18::pAWP78 (empty plasmid) were also removed. That allowed us to narrow the list to only three entries (FabF, WP_026130034.1; 3-hydroxydecanoyl-ACP dehydratase, WP_017839698.1; 2-isopropylmalate synthase, WP_017840721.1). Effects of up- and down-regulation of these genes will be tested in future studies.

**Table 5:** Opposite directions^*^ of differentially expressed genes in two datasets: original AP18 vs. down-regulated *farE* and AP18+pAWP78 compared to up-regulated *farE*.

	**Fold change**	
	**AP18 vs. AP18Δ*farE***	**AP18+pAWP78 vs. AP18::*farE***
**FATTY ACID METABOLISM**		
WP_026130034.1	0.9	2.5
3-Oxoacyl-ACP synthase (fabF)		
WP_014148504.1^**^	1.3	1.9
ACP		
WP_017839573.1^***^	0.3	2.2
Beta-ketoacyl-ACP reductase (fabG)		
WP_017839572.1 ^***^	2.2	0.8
Malonyl CoA-ACP transacylase		
WP_017839571.1 ^***^	0.6	2.7
3-Oxoacyl-ACP synthase		
WP_017839570.1 ^***^	0.4	4.6
Phosphate acyltransferase		
WP_017839698.1	0.9	1.8
3-Hydroxydecanoyl-ACP dehydratase		
WP_017839697.1^**^	0.8	1.1
Beta-ketoacyl-ACP synthase I		
**Acetyl-CoA POOL**		
WP_017840721.1:	0.9	1.2
2-Isopropylmalate synthase		
WP_026130168.1^**^	1.4	0.6
CDP-diacylglycerol–serine O-phosphatidyl transferase		

* Down-regulated in AP18 vs. AP18ΔfarE and up-regulated in AP18+pAWP78 vs. AP18::fare;

** *Neighboring gene*;

*** *Neighboring genes from one gene cluster*.

## Discussion

Microbial fatty acid biosynthesis pathways are among the most attractive targets for the production of fuels and oleochemicals from renewable feedstocks (Lennen and Pfleger, 2013; Janßen and Steinbuchel, 2014). Different microbial systems as well as numerous metabolic engineering strategies have been explored for improving biofuel production (Lennen and Pfleger, 2013). The most common approaches aim to reduce the catabolism of fatty acids (e.g., deletion of *fadBAE* or *fadR* genes), to eliminate feedback inhibition in the biosynthetic pathway (e.g., overexpression of *accABCD*, fabD with/without thioesterase I (*tesA*) genes and/or deletion of *fabR*), as well as incorporating heterologous enzymes or metabolic pathways for converting the FA intermediates into free fatty acids, fatty acid methyl esters, and fatty acid ethyl esters, olifins, etc. (Clomburg and Gonzales, 2010; Lennen and Pfleger, 2013; Janßen and Steinbuchel, 2014).

Core metabolic functions of many methanotrophic bacteria are interlinked with the development of intracytoplasmic membranes. As a result, methanotrophic bacteria have relatively high lipid/biomass content (9–10% of dry weight, mostly as phospholipids). Here, we investigated fatty acid biosynthesis in *M. buryatense 5G(B1)*. Overall, the metabolic pathways for fatty acid production and degradation in *M. buryatense 5G(B1)* resemble the canonical bacterial pathways with only a few exceptions, such as the lack of recognizable *fadD*/*fadL* and regulators. Absence of the *fadL* gene is not surprising since *M. buryatense 5G(B1)* is known for not being able to utilize long-chain FAs as a carbon or energy source. On the other hand, the absence of *fadD*, the mechanism by which free FAs becomes activated (i.e., ligation to CoA) for beta oxidation is puzzling. The phenotype of *fadABE* mutants indicates that beta-oxidation pathway is active in *M. buryatense 5G(B1)*. A set of genes annotated as Acyl-CoA synthetases were identified in the genome, each of them might potentially fulfill the *fadD* function.

Given that intra-cytoplasmic membranes are primarily produced during growth on methane and that a membrane-bound methane monooxygenase, pMMO, is associated with these membranes, a regulatory mechanism must exist to repress FA biosynthesis during growth on non-methane carbon sources (i.e., methanol). The initial hypothesis posed that *farE* could encode a FA metabolism regulator. Our results support this hypothesis. *farE* deletion changes expression of many metabolic genes including several involved in FA biosynthesis; the FA content of the mutant is also drastically changed. The phenotypic feature of AP18Δ*farE*, i.e., conversion of a significant portion of C16 FA into C18 FA, could be potentially explained by overproduction of 3 FA synthesis elongation enzymes, two *fabF* genes and one less characterized 3-ketoacyl-ACP synthase gene (WP_017841167.1). Another option for accumulation of C18 FAs could be overregulated *acpP* genes. Only one of the 3 *acpP* genes is highly transcribed (WP_014148504.1); the two others are transcribed in low amounts. However, these low-expressed copies of acyl carrier proteins could have different properties from the main one resulting in synthesis of longer FAs. It has been recently demonstrated that the size of the Acp hydrophobic pocket influences the length of FA synthesized, with smaller pockets leading to cellular accumulation of shorter FAs (Liu et al., 2016). In our case we would expect that one of the Acp copies would have a bigger hydrophobic pocket resulting in synthesis of longer lipids. In addition, FarE contributed to upregulation of a number FA biosynthesis genes, including *fabA* (hydroxyacyl-ACP dehydratase), *fabG* (3-ketoacyl-ACP reductase), and thioesterase. Overall, it could be suggested that FarE acts as a regulator of fatty acid metabolism, although there is a possibility that the observed AP18Δ*farE* phenotype could be achieved through indirect action of unidentified regulatory mechanisms.

A set of different strategies was tested for improving fatty acid production in *M. buryatense 5G(B1)*. While the overexpression of the *fabB* did not alter the FA composition significantly, the modulation of FA synthesis elongation seems to be a viable strategy to manipulate the FA content in methanotrophs. Analysis of transcriptomics data of the AP18Δ*farE* strain suggested three potential candidates for synthesis of longer-than-usual C18 FA in *M. buryatense 5G(B1)*. These proteins with 3-ketoacyl-ACP synthase (KAS) activities comprise 2 *fabF* genes and one previously unidentified KAS (WP_017841167.1) gene. Previously, *fabF* was identified as an activator of FA biosynthesis in *E. coli* (Lee et al., 2013), which makes it attractive target for biotechnological manipulation. Another difference is the ability of *fabF* but not *fabB* to elongate palmitoleic acid (C16:1) for synthesis of a C18:1 version (Edwards et al., 1997). However, whether the increased activity of *fabF* would be sufficient for switching of FA biosynthesis from predominantly C16 FA to C18 remains a question. In this regard, the third KAS enzyme (WP_017841167.1), which is still uncharacterized, could also be considered. Whether overexpression of any of them would result in changes in FA profile will be accessed in future studies.

It has been previously observed that overexpression of *acpP* from a multi-copy plasmid leads to inhibition of cell growth in *E. coli* (Keating et al., 1995). That effect was attributed to an unmodified (Apo) form of acyl carrier protein since overexpressed ACP was incompletely post-translationally modified and accumulation of apo-Acp resulted in an inability to transfer the completed FA to glycerol 3-phosphate. However, lower levels of Acp, including AcpS from other organisms, could be produced in *E. coli* without toxicity (Guerra et al., 1988). Since the overexpression plasmid used in the current study (pAWP78, Puri et al., 2015) is a medium- to low-copy number plasmid, we did not expect inhibitory effects of Acp overproduction in *M. buryatense 5G(B1)*. However, adverse effects resulting in inhibition of growth upon overproduction of Acp were observed indicating that the mechanism of post-translation modification of Acp by holo-ACP synthase is adjusted to regulate Acp levels and might be inefficient when Acp is overproduced. Further attempts to upregulate FA accumulation by Acp overproduction should be supported by simultaneous overproduction of holo-ACP synthase.

The genetic alterations of fatty acid turnover as well as key elements of fatty acid biosynthesis resulted in very minor changes in overall lipid content, suggesting the capacity of the system is restricted by the overall influx of carbon into the pathway. However, strains with *ack* deleted (block in acetyl-CoA to acetate conversion) showed an increased FA content. Moreover, a strain with both Δ*ack* and overexpression of *acc* had the highest FAME content among all tested in this study. The inconsistences in acc-overexpression outcomes could be explained by insufficient biotinylation of the protein.

## Summary

Metabolic pathways for fatty acid biosynthesis are reconstructed and a set of novel mechanisms contributing to FA-biosynthesis regulation and metabolic precursor formation are proposed. *M. buryatense 5G(B1)* naturally accumulates more FA than many other bacteria in order to maintain high amounts ICM structures (8.8% FAME content). In this work, several strategies were demonstrated to increase FA production in methanotrophic bacteria further (resulting up to 11% FAME content per dry cell weight). The tested strategies, despite targeting different non-overlapping pathways, were not usually additive suggesting the existence of a yet-to-be discovered mechanism of regulation of FA levels.

## Author contributions

AD, EA, and MK designed the experiments and coordinated the study. AD carried out all genetic, growth, and transcriptomic experiments and analyzed data. AD and MA performed genome mining and wrote the first draft of the manuscript. IA contributed to model construction and RNA-data analyses. MK and EA helped to analyze the data and write the manuscript. All authors reviewed and approved the final manuscript.

### Conflict of interest statement

The authors declare that the research was conducted in the absence of any commercial or financial relationships that could be construed as a potential conflict of interest.
