# Supplementary material for: Fatty Acid Biosynthesis Pathways in Methylomicrobium buryatense 5G(B1)
Source: Front Microbiol. 2017 Jan 10;7:2167. doi: 10.3389/fmicb.2016.02167 (PMC5222806; doi:10.3389/fmicb.2016.02167)
Supplement: Supplementary file 2 [file DataSheet2.docx]

**Table S1.** Growth medium composition**.**

| **Solution 1** | **g/L** | Comments |
| --- | --- | --- |
| KNO_3_ | 1 | Solution 1 was autoclave at 121^o^C for 30 minutes. |
| MgSO_4_ x 7H_2_O | 0.2 |  |
| CaCl_2_ x 2H_2_O | 0.02 |  |
| NaCl | 7.5 |  |
| Trace solution (1000x) | 1 ml |  |
| Water (distilled deionized water) | 1 L |  |
| **Agar** (solid media) | 2.2 g per 150 ml of Solution 1 | |
| **Phosphate solution** (pH 6.8) | 20 ml/L | Phosphate and carbonate solutions were prepared separately, and mixed with the medium just before inoculation. |
| **Carbonate solution**  (1M, pH 8.6-9.0) | 40 ml/L |  |
| **Trace solution (1000x)** |  | The solution was autoclaved at 121^o^C for 20 minutes and stored at room temperature for up to 6 months. |
| Na_2_EDTA | 5 |  |
| FeSO_4_ x 7 H_2_O | 2 |  |
| ZnSO_4_ x7 H_2_O | 0.3 |  |
| MnCl_2_ x 4 H_2_O | 0.03 |  |
| CoCl_2_ x 6 H_2_O | 0.2 |  |
| CuSO_4_ x 5 H_2_O | 1.2 |  |
| Na_2_O_4_W x 2H_2_O | 0.3 |  |
| NiCl_2_ x 6 H_2_O | 0.05 |  |
| Na_2_MoO_4_ x 2 H_2_O | 0.05 |  |
| H_3_BO_3_ | 0.03 |  |
| **Phosphate solution**  **(pH 6.8)** |  | Was autoclave at 121^o^C for 20 minutes. pH of the solution should be 6.8-7. |
| KH_2_PO_4_ | 5.44  5.68 |  |
| Na_2_HPO_4_ |  |  |
| **Carbonate solution (1M, pH 8.8-9.0)** |  | Was sterilized by filtration only. pH of the solution should be 8.6-9.0. |
| NaHCO_3_ | 75.6 |  |
| Na_2_CO_3_ | 10.5 |  |

**Table S2.** Sequences cloned to plasmids in this study (start/end nucleotide positions are shown according to NCBI RefSeq accession number NZ_KB455575.1

| Sequences Cloned | from, nt | to, nt |
| --- | --- | --- |
|  |  |  |
| Flanks for deletion of *fadABE* |  |  |
| upstream | 3990525 | 3991081 |
| downstream | 3983903 | 3984492 |
| Flanks for deletion of *farE* |  |  |
| upstream | 1083904 | 1084738 |
| downstream | 1082313 | 1083146 |
| ORFs for overexpression |  |  |
| *farE* | 1083258 | 1083842 |
| *fabB* | 1247063 | 1248283 |
| *acpP* | 1088519 | 1088749 |

**Table S3.** Transcriptomics data for AP18, AP18*ΔfarE,* AP18::*fare and* AP18::pAWP78 (empty plasmid) [attached Exl. File].

**Table S5.** Enzymes involved in pathways of the methane oxidation and FA synthesis in *M. buryatense 5G(B1)*.

| **Enzyme** | **Gene name** | **NCBI-Protein** |
| --- | --- | --- |
| EC 1.14.18.3  particulate methane monooxygenase | METBU_RS0118245  METBU_RS0118250  METBU_RS0118255 | WP_017840377.1  WP_017841993.1  WP_017841994.1 |
| EC 1.1.1.244 or EC 1.1.2.7  methanol dehydrogenase | METBU_RS0119465  METBU_RS0119480 | WP_017842205.1  WP_017842208.1 |
| EC 4.2.1.147  5,6,7,8-tetrahydromethanopterin hydrolyase | METBU_RS0100960  METBU_RS0101990  METBU_RS0105010  METBU_RS0106160 | WP_017838841.1  WP_017839027.1  WP_017839587.1  WP_014148886.1 |
| EC 1.5.1.-  methylene tetrahydromethanopterin dehydrogenase | METBU_RS0109120  METBU_RS0109125 | WP_017840326.1  WP_017840327.1 |
| EC 3.5.4.27  methenyltetrahydromethanopterin cyclohydrolase | METBU_RS0106145 | WP_014148883.1 |
| [no EC number assigned]  formyltransferase/hydrolase complex | METBU_RS0106335  METBU_RS0106340  METBU_RS0106365  METBU_RS0106370 | WP_017839816.1  WP_026130072.1  WP_017839819.1  WP_017839820.1 |
| EC 1.2.1.2  formate dehydrogenase | METBU_RS0103790  METBU_RS0113865  METBU_RS0113870  METBU_RS0113875  METBU_RS0113880 | WP_017839363.1  WP_017841204.1  WP_017841205.1  WP_017841206.1  WP_017841207.1 |
| EC 4.1.2.43  3-hexulose-6-phosphate synthase | METBU_RS0115890  METBU_RS0115910  METBU_RS0115930 | WP_017841571.1  WP_017841571.1  WP_017841571.1 |
| EC 5.3.1.27  3-hexulose-6-phosphate isomerase | METBU_RS0115895  METBU_RS0115915  METBU_RS0115935 | WP_017841572.1  WP_017841572.1  WP_017841572.1 |
| EC 2.7.1.90  pyrophosphate-fructose 6-phosphate 1-phosphotransferase | METBU_RS0111170 | WP_017840709.1 |
| EC 4.1.2.13  fructose-bisphosphate aldolase | METBU_RS0108720  METBU_RS0115960  METBU_RS0116805 | WP_017840254.1  WP_017841578.1  WP_026130284.1 |
| EC 5.3.1.1  triose-phosphate isomerase | METBU_RS0105770 | WP_026130060.1 |
| EC 1.2.1.12  glyceraldehyde-3-phosphate dehydrogenase | METBU_RS0122480  METBU_RS0117885 | WP_017842765.1  WP_017841928.1 |
| EC 2.7.2.3  phosphoglycerate kinase | METBU_RS0118680 | WP_017842070.1 |
| EC 2.7.1.165  glycerate 2-kinase | METBU_RS0110755 | WP_017840633.1 |
| EC 5.4.2.12  phosphoglyceromutase | METBU_RS0105465 | WP_017839666.1 |
| EC 4.2.1.11  enolase | METBU_RS0102020 | WP_017839033.1 |
| EC 2.7.1.40  pyruvate kinase | METBU_RS0101630  METBU_RS0122475 | WP_017838959.1  WP_017842764.1 |
| EC 5.3.1.9  glucose-6-phosphate isomerase | METBU_RS0114795 | WP_017841372.1 |
| EC 1.1.1.49  glucose-6-phosphate dehydrogenase | METBU_RS0115485  METBU_RS0116285 | WP_017841495.1  WP_017841635.1 |
| EC 3.1.1.31  6-phosphogluconolactonase | METBU_RS0116225 | WP_017841625.1 |
| EC 4.2.1.12  phosphogluconate dehydratase | METBU_RS0109185 | WP_017840337.1 |
| EC 4.1.2.14  2-dehydro-3-deoxy-phosphogluconate aldolase | METBU_RS0109190 | WP_017840338.1 |
| EC 4.1.2.22  fructose-6-phosphate phosphoketolase | METBU_RS0100495  METBU_RS0107415 | WP_017838760.1  WP_017840010.1 |
| EC 2.3.1.8  phosphate acetyltransferase | METBU_RS0109465 | WP_017840391.1 |
| EC 1.2.1.-  pyruvate dehydrogenase | METBU_RS0109200  METBU_RS0109205 | WP_017840340.1  WP_017840341.1 |
| EC 6.3.4.3  formate—tetrahydrofolate ligase | METBU_RS0110765 | WP_017840634.1 |
| EC 3.5.4.9  methenyltetrahydrofolate cyclohydrolase | METBU_RS0119010  METBU_RS0108920 | WP_017842126.1  WP_017840290.1 |
| EC 1.5.1.5  methylenetetrahydrofolate dehydrogenase | METBU_RS0110750  METBU_RS0108920 | WP_014149646.1  WP_017840290.1 |
| EC 2.1.2.1  glycine hydroxymethyltransferase | METBU_RS0110760 | WP_014149648.1 |
| EC 2.6.1.45  serine—glyoxylate transaminase | METBU_RS0110700 | WP_017840623.1 |
| EC 4.1.3.24  malyl-CoA lyase | METBU_RS0110690 | WP_017840621.1 |
| EC 2.7.2.1  acetate kinase | METBU_RS0107420 | WP_017840011.1 |
| EC 6.4.1.2  acetyl-CoA carboxylase | METBU_RS0102130  METBU_RS0109450  METBU_RS0102635  METBU_RS0102640  METBU_RS0119405 | WP_017839055.1  WP_017840388.1  WP_017839151.1  WP_017839152.1  WP_017842193.1 |
| EC 2.3.1.39  malonyl CoA-ACP transacylase | METBU_RS0104930 | WP_017839572.1 |
| EC 2.3.1.180  3-oxoacyl-ACP synthase | METBU_RS0104925 | WP_017839571.1 |
| EC 1.1.1.100  3-ketoacyl-ACP reductase | METBU_RS0113640  METBU_RS0104935 | WP_017841165.1  WP_017839573.1 |
| EC 4.2.1.59  3-hydroxyacyl-ACP dehydratase | METBU_RS0113645  METBU_RS0110470 | WP_017841166.1  WP_014149549.1 |
| EC 1.3.1.9  enoyl-ACP reductase | METBU_RS0100175 | WP_014149471.1 |
| EC 2.3.1.41  3-oxoacyl-ACP synthase | METBU_RS0104165  METBU_RS0105635  METBU_RS0113635 | WP_017839429.1  WP_017839697.1  WP_026130222.1 |
| EC 3.1.2.14  lipase | METBU_RS0117145 | WP_040575815.1 |
| EC 3.1.2.2  acyl-CoA thioester hydrolase | METBU_RS0108930 | WP_040575583.1 |
| EC 1.1.1.94  glycerol-3-phosphate dehydrogenase | METBU_RS0112685 | WP_017840981.1 |
| EC 2.3.1.15  glycerol-3-phosphate 1-O-acyltransferase | METBU_RS0114885  METBU_RS0104920 | WP_017841388.1  WP_017839570.1 |
| EC 1.3.8.-  acyl-CoA dehydrogenase | METBU_RS0117915 | WP_017841932.1 |
| EC 4.2.1.17  enoyl-CoA hydratase | METBU_RS0117920 | WP_017841933.1 |
| EC 1.1.1.35  3-hydroxyacyl-CoA dehydrogenase | METBU_RS0117920 | WP_017841933.1 |
| EC 2.3.1.16  3-ketoacyl-CoA thiolase | METBU_RS0117925 | WP_017841934.1 |
